# A comprehensive genome-wide analysis of long non-coding RNA and mRNA expression profiles of JAK2V617F-positive classical myeloproliferative neoplasms

**DOI:** 10.1080/21655979.2021.2000226

**Published:** 2021-12-14

**Authors:** Jie Zhou, Hao Wu, Cheng Guo, Bing Li, Li-Li Zhou, Ai-Bin Liang, Jian-Fei Fu

**Affiliations:** a Tongji University School of Medicine, Shanghai, 200092, China; b Department of Gastroenterology, Tongji Hospital of Tongji University, Shanghai, 200065, China; c Department of Hematology, Tongji Hospital of Tongji University, Shanghai, 200065, China

**Keywords:** Classical myeloproliferative neoplasms, JAK2V617F-positive, LncRNA, mRNA, microarray analysis, ITGB3

## Abstract

Aberrant expression of long non-coding RNAs (lncRNAs) is involved in the progression of myeloid neoplasms, but the role of lncRNAs in the JAK2V617F-positive subtype of classical myeloproliferative neoplasms (cMPNs) remains unclear. This study was conducted to clarify the expression and regulation patterns of lncRNAs in JAK2V617F-positive cMPNs, and to explore new potential carcinogenic factors of cMPNs. Bioinformatics analysis of microarray detection and wet testing verification were performed to study the expression and regulation signature of differentially expressed lncRNAs (DELs) and related genes (DEGs) in cMPNs. The expression of lncRNAs and mRNAs were observed to significantly dysregulated in JAK2V617F-positive cMPN patients compared with the normal controls. Co-expression analysis indicated that there were significant differences of the co-expression pattern of lncRNAs and mRNAs in JAK2V617F-positive cMPN patients compared to normal controls. GO and KEGG pathway analysis of DEGs and DELs showed the involvement of several pathways previously reported to regulate the pathogenesis of leukemia and cMPNs. Cis- and trans-regulation analysis of lncRNAs showed that ZNF141, DHX29, NOC2L, MAS1L, AFAP1L1, and CPN2 were significantly cis-regulated by lncRNA ENST00000356347, ENST00000456816, hsa-mir-449c, NR_026874, TCONS_00012136, uc003lqp.2, and ENST00000456816, respectively, and DELs were mostly correlated with transcription factors including CTBP2, SUZ12, REST, STAT2, and GATA4 to jointly regulate multiple target genes. In summary, expression profiles of lncRNAs and mRNAs were significantly altered in JAK2V617F-positive cMPNs, the relative signaling pathway, co-expression, cis- and trans-regulation were regulated by dysregulation of lncRNAs and several important genes, such as ITGB3, which may act as a promising carcinogenic factor, warrant further investigation.

## Introduction

Philadelphia chromosome-negative classic myeloproliferative neoplasms (cMPNs), are hematological clonal disorders characterized by the hyperplasia of mature myeloid cell lineages [1]. The pathogenesis of the disease involves a multifactorial process characterized by the recurrent somatic mutations in genes such as JAK2, CALR, and MPL [2]. These driver mutations cause the cytokine-independent activation of the JAK-STAT pathway and thus initiates the cMPNs [3]. The G to T somatic mutation at nucleotide 1849, in exon 14 of JAK2, resulting in the substitution of valine to phenylalanine at codon 617 (JAK2V617F) in the pseudokinase domain [4], can be found in around 70% of MPNs. JAK2V617F-positive cMPN is one of the most common subtypes of the cMPN and the vast majority of cMPN patients are JAK2V617F-positive MPNs.

Long non-coding RNA (lncRNAs) are RNA molecules that are more than 200 nucleotides in length which do not code any protein [5]. Several studies have shown that lncRNAs are intermediaries between DNA and protein as well as protagonists of cellular functions [6]. LncRNAs are reported to alter gene expression by transcription and post-transcriptional processing, as well as by chromosome remodeling [7]. In addition, dysregulation of lncRNAs is increasingly reported to be associated with human cancers. Studies have shown that aberrant expression of lncRNAs is associated with the progression of myeloid tumors.Yildirim et al [8]. demonstrated that deletion of lncRNA X–inactive specific transcript in the blood compartment of mice led to the genome-wide changes followed by mixed MPN/MDS In BCR-ABL-mediated chronic myeloid leukemia (CML), lncRNA BGL3 binds microRNAs to inhibit phosphatase and tensin homolog (PTEN) expression, and silencing of tumor-suppressor lncRNA-BGL3 promotes BCR-ABL-mediated cellular transformation [9]. In addition, overexpression of lncRNA-H19 promotes CML progression by increasing cell survival and inhibiting apoptosis [10]. However, the regulatory pattern lncRNAs involved in the pathogenesis of cMPNs remains unclear.

We proposed the hypothesis that the expression profiles of lncRNAs and mRNAs were dysregulated in cMPN patients compared with normal controls, and the aims and goals of the present study were to explore the differences of lncRNA and mRNA expression profiles of the JAK2V617F-positive subtype of cMPNs compared to normal individuals and to investigate the potential roles of lncRNAs in pathogenesis of JAK2V617F-positive cMPNs. The expression patterns or profiles of lncRNAs and mRNAs in JAK2V617F-positive cMPNs were evaluated by microarray technology. Bioinformatics analysis were used to study the expression signature of differentially expressed genes (hereinafter referred to as DEGs) and differentially expressed lncRNAs (hereinafter referred to as DELs) in JAK2V617F-positive cMPNs patients compared with normal controls. We validated the top DELs and some of the identified DEGs according to the microarray analysis and enriched the co-expressed mRNAs to predict the regulatory roles of lncRNAs in cMPNs. Gene Ontology (GO) and Kyoto Encyclopedia of Genes and Genomes (KEGG) databases were used to obtain a better annotation of the biological roles of DELs and DEGs. Furthermore, cis- and trans-regulation analyses of lncRNAs were performed to determine the potential target genes and transcription factors (TFs) associated with the DELs to identify TFs or chromatin regulators that may play a regulatory role in conjunction with lncRNAs in JAK2V617F-positive cMPNs. Our findings provide complementary understanding of the roles of lncRNAs in cMPNs and shed light on potential novel strategies for diagnosis and therapy.

## Materials and methods

### Patients and clinical samples

A total of 12 samples (six from JAK2V617F-positive cMPNs patients and six from healthy normal controls) were included in our study. All patients were from the Tongji hospital of Tongji University. The diagnosis of cMPNs was based on diagnostic criteria according to the WHO [11]. The acquisition and detection of these clinical samples was performed according to the Declaration of Helsinki and was approved by the medical ethic committee of Tongji hospital of Tongji University (Number: 2021-KYSB-177). Written informed consent was obtained from each participant. Clinical characteristics of six JAK2V617F-positive cMPNs patients and six normal controls, from which bone marrow mononuclear cells were isolated, were shown in Table 1. Total RNA was extracted from bone marrow mononuclear cells using TRIzol reagent [12] (Invitrogen, Carlsbad, CA, USA).

**Table 1.** Clinical characteristics of 12 samples (six JAK2V617F-positive cMPNs patients and six normal controls), from which bone marrow mononuclear cells were isolated

No.	JA2V617F Mutation	MPL Mutation	CALR Mutation	Sex	Age (years)	Diagnosis	White blood cell (×10^9/L)	Platelet count (×10^9/L)	Hemoglobin (g/L)	Ferritin (μg/L)	Enlarged liver or spleen (mm×mm)	Karyotype
1	Negative	Negative	Negative	Male	76	-	5.8	140	124	357	-	Normal
2	Negative	Negative	Negative	Male	61	-	4.1	180	135	71.9	-	Normal
3	Negative	Negative	Negative	Female	58	-	3.4	130	121	140.5	-	Normal
4	Negative	Negative	Negative	Male	45	-	5.62	157	152	215	-	Normal
5	Negative	Negative	Negative	Female	53	-	2.87	276	112	508	-	Normal
6	Negative	Negative	Negative	Female	34	-	2.84	290	148	342	-	Normal
7	Positive	Negative	Negative	Female	63	PV	12.74	575	132	12.63	Splenomegaly, (43 × 150)	Normal
8	Positive	Negative	Negative	Male	65	PV	10.33	287	195	975.5	Splenomegaly, (44 × 112)	Normal
9	Positive	Negative	Negative	Female	83	ET	11.09	377	100	173	Splenomegaly, (99 × 269)	The two cells have abnormal structures on chromosomes 7 and 18 respectively, but do not form clones.
10	Positive	Negative	Negative	Male	71	ET	21.43	797	142	130	Splenomegaly, (53 × 180)	One cell showed a polyploid karyotype, with structural abnormalities on chromosome 19.
11	Positive	Negative	Negative	Male	64	PMF	44.78	160	118	557	Splenomegaly, (53 × 190)	Normal
12	Positive	Negative	Negative	Female	59	PMF	10.8	158	129	126	Splenomegaly, (77 × 277)	46,XX,add(3)(q12)[5]/46,XX[15]

### Cell lines and culture

The human erythroleukemia cell line HEL, human immortalized myelogenous leukemia cell line K562, human Caucasian bone marrow acute myelogenous leukemia cell line KG1α, human myeloid leukemia cell lines U937, NB4, THP1, and MV4-11 were purchased from the Cell Bank, Chinese Academy of Sciences (Shanghai, China) and were characterized using Short Tandem Repeat markers [13]. HEL, U937, NB4, MV4-11, and THP1 cells were grown in Roswell Park Memorial Institute (RPMI) 1640 Medium (Gibco; Thermo Fisher Scientific, Inc.). K562 and KG1α cells were grown in Iscove’s Modified Dulbecco’s Medium (IMDM, Gibco; Thermo Fisher Scientific, Inc.). All media were supplemented with 1% penicillin-streptomycin (Gibco; Thermo Fisher Scientific, Inc.) and 10% fetal bovine serum (FBS; Gibco; Thermo Fisher Scientific, Inc.). All these cell lines were cultured in a humidified atmosphere of 5% CO_2_ at 37°C and all were found to be negative for *Mycoplasma* contamination.

### Microarray analysis of lncRNA and mRNA expression patterns in cMPN patients and normal controls

The Agilent Array platform was employed for microarray analysis [14]. The sample preparation and microarray hybridization were performed based on the manufacturer’s standard protocols. The arrays were scanned by the Agilent Scanner G2565BA . Differentially expressed mRNA or lncRNA with an absolute value of fold change (FC) ≥ 1.5 and the false discovery rate (FDR) < 0.05 were considered statistically significant. In-depth mining of microarray data including pathway enrichment, co-expression analysis, cis-regulatory gene analyses, and trans-regulatory gene analysis were performed to better annotate the roles of lncRNAs and related-mRNAs in JAK2V617F-positive cMPNs.

### Quantitative real-time PCR (qRT-PCR) validation of DELs and DEGs

Several lncRNAs and mRNAs were selected for experimental validation using qRT-PCR. Total RNAs of independent samples from the 6 JAK2V617F-positive cMPN patients and 6 normal controls whom were included in the microarray analysis were extracted using TRIzol. The primer sequences of the dysregulated mRNAs are listed in Table 2, and the primer sequences of the dysregulated lncRNAs are listed in Table 3. The genes GAPDH and β-actin were chosen to be the endogenous reference genes for normalization [15]. The ΔΔCt method was used for relative quantification of gene expression [16].

**Table 2.** Primers for qRT-PCR for validation of the expression of DEGs

Name	Primer	Sequence	Product size
SOCS2	Forward	TTAAAAGAGGCACCAGAAGGAAC	199bp
	Reverse	AGTCGATCAGATGAACCACACT	
ESAM	Forward	CCAACTTGCTGCGGTTTTTGT	248bp
	Reverse	TGTGACCCCATTGATGTAGGA	
FCGR1A	Forward	TGGCCTTGAGGTGTCATGC	297bp
	Reverse	GCAAGAGCAACTTTGTTTCACA	
ITGB3	Forward	GTGACCTGAAGGAGAATCTGC	161bp
	Reverse	CCGGAGTGCAATCCTCTGG	
KIR3DL1	Forward	CGTGTGTTGGGTTGTTCTTG	225bp
	Reverse	GGCTCATGTTGAAGCTCTCC	
FGF2	Forward	AGTGTGTGCTAACCGTTACCT	77bp
	Reverse	ACTGCCCAGTTCGTTTCAGTG	
GAPDH	Forward	GGAGCGAGATCCCTCCAAAAT	197bp
	Reverse	GGCTGTTGTCATACTTCTCATGG

**Table 3.** Primers for qRT-PCR for validation of the expression of DELs

Name	Primer	Sequence	Product size
ENST00000356347	Forward	ACCAGAGAATTCAGACAGGT	92bp
	Reverse	ATTTGCTGTTTAAAGCCAAAGG	
ENST00000456816	Forward	TCCTGGCCTTTGCAGTTA	133bp
	Reverse	GTCATGGGAATGGAATGTCT	
TCONS_00012136	Forward	TCTGGGTGTAACCTTTCAGATT	95bp
	Reverse	CAGCATGTGCCAGAACTT	
ENST00000452247	Forward	AGTGCTGAGATTACAAGTGTGA	171bp
	Reverse	GGCAAGAGAAATCTATGTGGTC	
uc002wvw.2	Forward	GTAAATGTGGCTGGTCCTTG	82bp
	Reverse	TGCCTGAAGAATGAAAGGGT	
uc003nog.1	Forward	TCTTTGTCCCTGGTTTCTCC	85bp
	Reverse	CAGCCAAGAGTCTTCTCAT	
uc001dvp.2	Forward	AAGGAGTAAAGCAACTTCCAT	89bp
	Reverse	TCACCAACCTCTTGGGAATC	
ENST00000425630	Forward	CACAGAGCACTGCATTGG	89bp
	Reverse	GCCTAGCTCTGTCTCATTTG	
β-actin	Forward	CATTCCAAATATGAGATGCGTT	133bp
	Reverse	TACACGAAAGCAATGCTATCAC	

### Western blotting

Total protein was extracted using the RIPA buffer (PC101, Epizyme, Inc., Cambridge, MA, USA) containing a protease inhibitor cocktail (P1005, Beyotime, Beijing, China). The BCA method (P0011, Beyotime) was used to detect the protein concentration. Total proteins (20 μg) were separated by 10% SDS-PAGE and transferred onto PVDF membranes (Millipore, Billerica, MA, USA). The membranes were probed with antibodies (18,309-1-AP for ITGB3, 17,670-1-AP for JAK2, and 66,009-1-Ig for β-actin from Proteintech, China). The protein bands were detected with an Amersham Imager 600 (GE Healthcare, Waukesha, WI, USA).

### LncRNA-mRNA correlation analysis

Among the upregulated- and downregulated-DELs, the top 200 (400 in total) coding genes for each group were selected among those satisfying the p < 0.05 cutoff and the raw data of mRNA microarray analysis were included for further lncRNA-mRNA correlation analysis. For each selected lncRNA, the Pearson’s correlation with each mRNA was calculated according to the expression level, the absolute value of which not less than 0.8 and the p-value of which not higher than 0.05 were used as cutoff values to obtain the most probable biologically relevant lncRNA-mRNA regulation pairs. The ‘base’ function and ‘stat’ packages in the R (www.r-project.org/) environment [17] were used to preprocess data and calculate Pearson’s correlation coefficient. Correlated expressed mRNAs for the lncRNAs was identified based on the lncRNA-mRNA correlation analysis as previously described [18]. In addition, we took the most relevant 30 mRNA expression values and lncRNA expression values for unsupervised cluster analysis and drew a heatmap using the ‘heatmaps’ [19] package in the R.

### Functional prediction of lncRNAs and enrichment analysis of differentially expressed genes

For function prediction of mRNAs, the GO and KEGG enrichment analyses of the identified DEGs were performed using DAVID (https://david.ncifcrf.gov/) [20]. A previously described method was adopted for function prediction of these differentially expressed lncRNAs [21]. In brief, the enriched functional terms of co-expressed DEGs for each DELs were used as the functional terms for the corresponding lncRNA [22]. The top 200 prediction pairs with the highest prediction reliability (sorted by p-value) were selected, and the frequency of each function prediction term was counted, and the GO (or KEGG pathway) terms with a greater number of annotations were adopted to reflect differences obtained in the study for the overall function of lncRNAs, and selected the top30 (counted by frequency) to construct a bar graph.

### Cis-regulatory gene analyses for the dysregulated lncRNAs in JAK2V617F-positive cMPNs

For each lncRNA, we defined mRNAs as ‘cis-regulated mRNAs’ [23] when the mRNAs loci were within the 300k windows up- and downstream of the given lncRNA, and when the Pearson’s correlation coefficient of lncRNA-mRNA expression is significant (P-value of correlation ≤0.05).

### Trans-regulatory gene analyses for the dysregulated lncRNAs in JAK2V617F-positive cMPNs

Based on the analysis of the co-expressed mRNA for each differentiated lncRNAs, the enrichment significance of the DEGs of each TF was calculated according to the hypergeometric distribution, and the p-value≤0.05 indicated that the difference gene expression was enriched in the TF entry. Next, we performed the trans-regulatory gene analyses to explore the lncRNAs-TFs network in JAK2V617F-positive cMPNs, and the network was drawn by Cytoscape 3.01 software [24].

### Statistical analysis

Student’s t-test were performed to compare microarray and qRT-PCR assay results. FC ≥ 1.5 and P < 0.05 were considered statistically significant. Data are represented as the mean ± standard deviation (SD) of more than three independent experiments.

## Results

In our study, we mainly explored the dysregulation of expression profiles of lncRNAs and mRNAs in JAK2V617F-positive cMPNs. We proposed the hypothesis that the expression profiles of lncRNAs and mRNAs were dysregulated in JAK2V617F-positive cMPN patients compared with normal controls, and the aims and goals of the present study were to investigate the potential roles of lncRNAs and mRNAs in pathogenesis of JAK2V617F-positive cMPNs. Bioinformatics analysis of microarray detection and wet testing verification were performed to study the expression and regulation signature of DELs and DEGs in JAK2V617F-positive cMPNs. Co-expression analysis was also performed to study the co-expression pattern of lncRNA and mRNA in JAK2V617F-positive cMPN patients. GO and KEGG pathway analysis of DEGs and DELs were performed to show the involvement of pathways in JAK2V617F-positive cMPNs, which were previously reported to regulate the pathogenesis of leukemia and cMPNs. Cis- and trans-regulation analysis of lncRNAs were performed to explore the mechanism of how these aberrant lncRNAs realize functions through cis- or trans-regulating mRNAs and to establish a three-element network correlation among lncRNA-TFs-target genes, which might participate in the pathogenesis of JAK2V617F-positive cMPNs.

### Identification of differently-expressed lncRNAs and mRNAs

**Figure 1.**
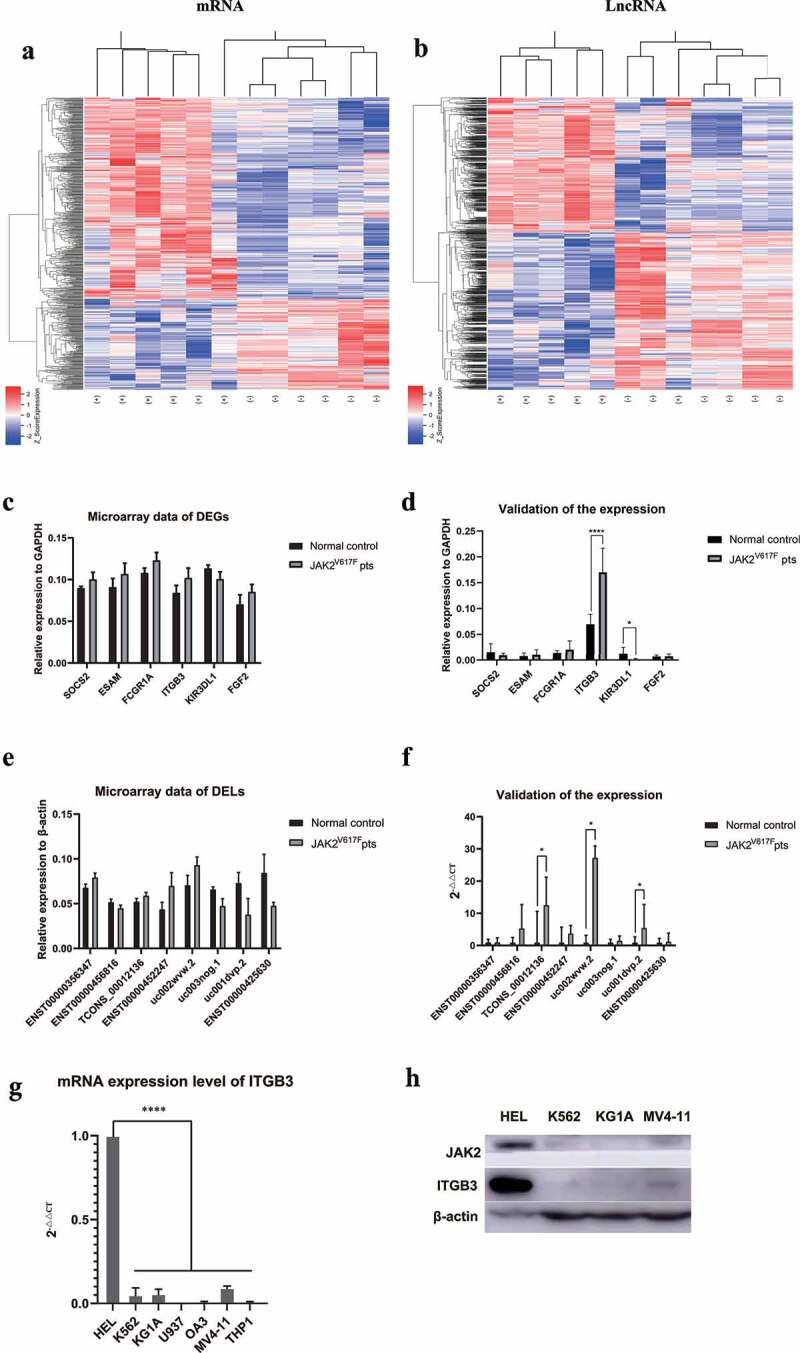
The lncRNA and mRNA expression profiles of 12 samples (six JAK2V617F-positive cMPNs patients and six JAK2V617F-negative normal controls). (a) Expression of mRNA between JAK2V617F-positive cMPNs patients and JAK2V617F-negative normal controls. (b) Expression of lncRNA between JAK2V617F mutation-positive MPN patients and mutation-negative controls. (c) Microarray data of differently expressed genes. (d) Validation of the expression of differently expressed genes by qRT-PCR. (e) Microarray data of differently expressed lncRNAs. (f) Validation of the expression of differently expressed lncRNAs by qRT-PCR. (g) Transcription levels of ITGB3 in myeloid leukemia cell lines. (h) Translation levels of ITGB3 in myeloid leukemia cell lines. (*P < 0.05; ****P < 0.0001)

A genome-wide microarray analysis were performed on six JAK2V617F-positive cMPNs patients and six normal controls, and the results showed that there were series of DELs and DEGs between the two subsets. Among the top 200 DELs and DEGs (400 in total), 112 lncRNAs and 149 mRNAs were up-regulated while 88 lncRNAs and 51 mRNAs were down-regulated in JAK2V617F-positive cMPN patients. The heatmap analysis revealed that the expression of lncRNAs and mRNAs were significantly dysregulated in JAK2V617F-positive cMPN patients compared with the normal controls (Figures 1(a,b). The top 20 DEGs and DELs were summarized in Table 4 and Table 5. In order to provide new ideas for further research on lncRNAs in JAK2V617F-positive cMPNs by screening potential pathogenic factors based on the findings in this study, we selected SOCS2, ESAM, FCGR1A, ITGB3, KIR3DL1, and FGF2 (Figure 1(c)) among the top 200 DEGs to further validate the expression in cMPNs patients using qRT-PCR. Although these genes were not with the greatest difference in expression, they were still selected for qRT-PCR validation because of their potential roles in the pathogenesis of cMPNs and other hematologic malignancies [25–30]. The qRT-PCR validation results showed that the expression of ITGB3 increased in JAK2V617F-positive cMPN patients compared to normal controls, and the expression of KIR3DL1 decreased in JAK2V617F-positive cMPN patients compared to normal controls, which were consistent with the microarray data (Figure 1(d)). We found that there were no statistically significantly differences in expression of SOCS2, ESAM, FCGR1A, and FGF2 between the six JAK2V617F-positive cMPN patients and six normal control samples from validation results (Figure 1(d)). Similarly, eight of the most significantly DELs obtained in the microarray analysis (Figure 1(e)) were selected for further validation in cMPNs patients by qRT-PCR. The results showed that TCONS_00012136, uc002wvw.2, and uc001dvp.2 were significantly higher expressed in JAK2V617F-positive cMPN patients compared to their normal control counterparts, which was consistent with the microarray data (figure 1(f)). There were no statistically significantly differences in expression of ENST00000356347, ENST00000456816, ENST00000452247, uc003nog.1, and ENST00000425630 between the six JAK2V617F-positive cMPN patients and six normal control samples (figure 1(f)). Some of the discrepancies between the microarray results and the qRT-PCR validation results may be due to the small sample size, but the overall trend remained consistent.

**Table 4.** Top 20 dysregulated mRNAs detected using microarray assays

Upregulated mRNA	Fold-change	Downregulated mRNA	Fold-change
PAFAH1B2	2.335863917	CRH	−2.16108225
STAC	2.307890583	DEFB1	−1.843066917
OTOF	2.293541083	ODZ1	−1.657750533
IDO1	2.112260017	MMP13	−1.501206583
DOK6	2.0680684	NCR3	−1.467082833
PROS1	1.9692475	CLDN22	−1.436974917
CXCL5	1.864472517	SLC25A24	−1.422179583
FOXA2	1.809072483	NRCAM	−1.420518117
CACNA1G	1.794914117	EYS	−1.372055383
ITGB3	1.774453167	PRHOXNB	−1.35500755
PCSK6	1.760886567	KIR3DL1	−1.295622833
HIST1H4H	1.756565083	SPP1	−1.283550317
ARL17A	1.699794167	ODZ1	−1.24132785
PLOD2	1.690670033	FGD2	−1.238391333
GBP1	1.617399333	NOP56	−1.217746333
MMP1	1.617288383	CDO1	−1.18732425
HIST1H1C	1.585112667	SEC23IP	−1.178565167
LY6G6D	1.584221383	TBX5	−1.1733468
ESAM	1.564616333	TMEM26	−1.143501133
HBE1	1.502833833	LOXHD1	−1.115511283

**Table 5.** Top 20 dysregulated LncRNAs detected using microarray assays

Upregulated LncRNA	Fold-change	Downregulated LncRNA	Fold-change
ENST00000452247	6.1619534	ENST00000425630	−12.856851
ENST00000447039	3.3244889	uc001dvp.2	−11.555164
hsa-mir-30c-2	2.8957493	uc003nog.1	−3.6053221
ENST00000567390	2.7878993	NR_045196	−3.273322
ENST00000528381	2.7409742	ENST00000515247	−3.1126108
hsa-mir-4436b-1	2.603541	ENST00000505166	−2.8805928
ENST00000513378	2.5824766	NR_023926	−2.7485877
ENST00000438151	2.4951467	ENST00000426904	−2.7465746
ENST00000416168	2.492804	ENST00000420905	−2.6768777
ENST00000452037	2.4125012	ENST00000557891	−2.6763823
ENST00000418366	2.381844	ENST00000513381	−2.5980875
NR_045213	2.3728048	TCONS_00021914	−2.5532334
NR_033319	2.358547	hsa-mir-4324	−2.5071294
TCONS_00022110	2.345018	ENST00000503553	−2.5006404
ENST00000513037	2.3408008	ENST00000561165	−2.4727013
ENST00000256692	2.329604	ENST00000440151	−2.4557002
TCONS_00014627	2.323738	ENST00000579413	−2.4515312
TCONS_00000822	2.2997477	ENST00000427868	−2.4457963
ENST00000536341	2.275378	TCONS_00029142	−2.4334197
ENST00000431695	2.250881	TCONS_00005763	−2.4040209

Given that in JAK2V617F-positive cMPN patients, the transcription level of the ITGB3 gene is much higher than that of normal samples, we suspected that a similar phenomenon may be observed in the myeloid tumor cell lines. Thus, we selected a variety of myeloid tumor cell lines to evaluate ITGB3 transcription levels (Figure 1(g)). Among the selected myeloid tumor cell lines, the HEL cell line is a human erythroleukemia cell line with JAK2V617F mutation, which as shown in Figure 1(g), when the ITGB3 expression was compared to all other cell lines harboring wild type JAK2, it was markedly higher. Furthermore, we detected the protein level of ITGB3 in the HEL, K562, KG1A and MV4-11 cells, to verify the relationship between ITGB3 expression and JAK2V617F mutation at the protein level. As showed in Figure 1(h), the protein level of JAK2 is the highest in HEL, in which JAK2 gene was abnormally activated by JAK2V617F mutation. Correspondingly, it can be observed that the expression of ITGB3 in HEL cells was also highly elevated. The acquired somatic mutation of the tyrosine kinase JAK2 gene (JAK2V617F) is the most important pathogenesis of cMPNs [31]. Therefore, the aforementioned results further confirmed that in JAK2V617F-positive cells, ITGB3 was elevatedly expressed, and the mechanisms under which deserves further investigation might shed light on the study of pathogenesis and prognosis of JAK2V617F-positive cMPNs. The above results preliminarily showed that there are significant differences in the mRNA and lncRNA expression profiles of JAK2V617F-positive cMPN patients compared to the normal controls. In addition, ITGB3 may be an important target in JAK2V617F-positive cMPNs. Among the top 200 DELs, we chose the eight DELs with the greatest difference in expression to further validate their expression in JAK2V617F-positive cMPN patients using qRT-PCR. The results of the qRT-PCR validation were consistent with the microarray data. These results indicated that the mRNA and lncRNA expression profiles were significantly altered in JAK2V617F-positive cMPN patients compared to normal controls, and the in-depth study of the changes in expression profiles represented an effective approach to further elucidate the pathogenesis of JAK2V617F-positive cMPNs.

### Co-expression network analysis of DELs and DEGs

**Figure 2.**
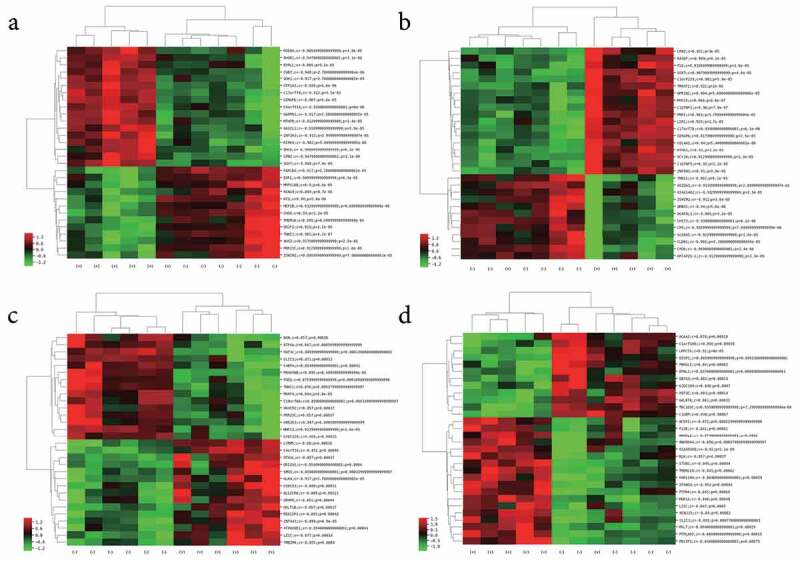
Co-expressed mRNAs heatmaps of differential expressed-lncRNAs. (a) lncRNA_ENST00000414065_cluster, (b) lncRNA_ENST00000427852_cluster, (c) lncRNA_ENST00000561476_cluster, (d) lncRNA_NR_023926_cluster

During the in-depth mining of microarray data, a lncRNA and mRNA co-expression network was constructed based on the correlation analysis in the present study. For each DEL, we calculated the Pearson’s correlation with the expression of each mRNAs detected in the microarray. Only DELs and DEGs with absolute values of Pearson’s correlation coefficient > 0.80 and the FDR value < 0.05 were considered correlated. A series of sets of DEGs correlated with each DEL, respectively. The top 30 correlated DEGs for each DEL were chosen to perform the unsupervised cluster analysis and constructed heatmaps (Supplementary Table 1). Figure 2 shows the heatmaps of four typical lncRNAs, lncRNA_ENST00000414065 (Figure 2(a)), lncRNA_ENST00000427852 (Figure 2(b)), lncRNA_ENST00000561476 (Figure 2(c)), and lncRNA_NR_023926 (Figure 2(d)), and their co-expressed top 30 mRNAs. The co-expression DEGs of all the four lncRNAs mentioned above showed significant differences between JAK2V617F-positive cMPN patients and normal controls, and indicated that there is significant alteration in the lncRNA regulatory system and the co-expression network of DELs and DEGs in JAK2V617F-positive cMPNs patients.

### Function prediction of differentially expressed mRNAs

**Figure 3.**
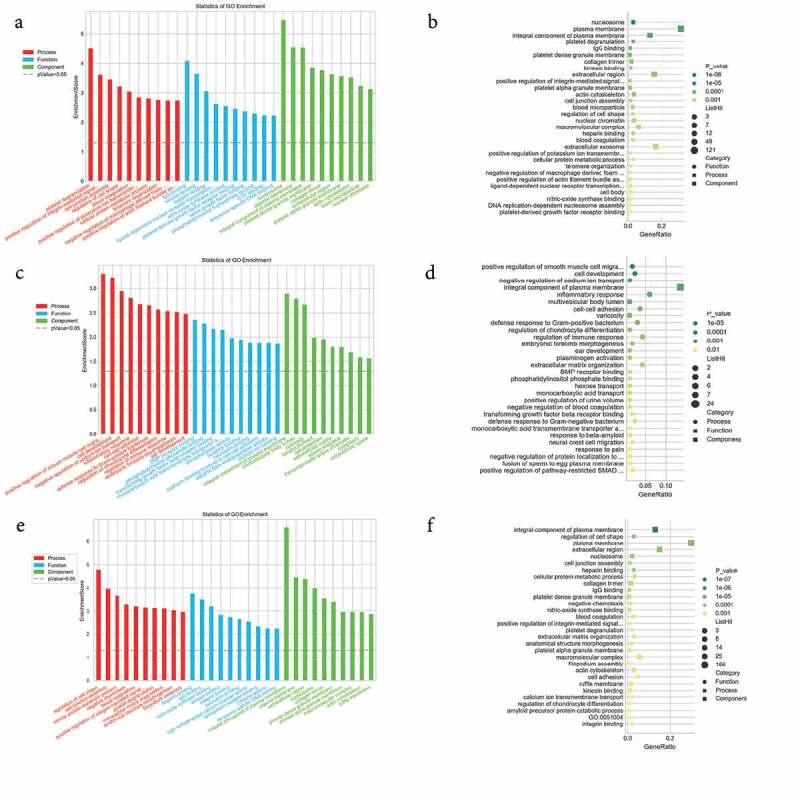
GO enrichment analysis of differentially expressed genes. (a) and (b) GO enrichment results of up-regulated differentially expressed genes (DEGs). (c) and (d) GO enrichment results of down-regulated DEGs. (e) and (f) GO enrichment results of all DEGs. In the bubble chart, the size of the dot represents the number of genes, and the color of the dot represents the p-value

To predict the potential biological roles of the DEGs in JAK2V617F-positive cMPN patients, GO and KEGG analyses were performed to explore the entire set of statistically significant DEGs. Based on the GO and KEGG analyses, we selected the reliably predicted terms having the top 10 frequency counts from the GO analysis data of all DEGs, upregulated DEGs and downregulated DEGs to construct bar and bubble charts (Figure 3). The GO analysis indicated that the top 10 enriched GO terms in the biological processes (BP), molecular functions (MF), and cellular components (CC) groups were all markedly different between the upregulated DEGs group (Figures 3(a,b)) and downregulated DEGs group (Figures 3(c,d)). But between the total DEGs GO analysis data (Figures 3(e,f)) and the upregulated DEGs group GO analysis data, the top 10 enriched GO terms were the same. For example, the most enriched biological process in upregulated DEGs group was the ‘platelet degranulation’ term, while in the downregulated DEGs group the most downregulated term was ‘positive regulation of smooth muscle cell proliferation’ term, while the ‘platelet degranulation’ term even was not on the list of the top 10 members of this group’s GO analysis data. The situation was similar in the molecular function and cellular component groups (Figure 3 and Supplementary Table 2). The only exception was the ‘integral component of plasma membrane’ term in the cellular component category. It appeared most often in the downregulated DEGs group and as the third in the upregulated DEGs group. ITGB3 was identified among the DEGs in the ‘integral component of plasma membrane’ terms and warrants further in-depth research.

**Figure 4.**
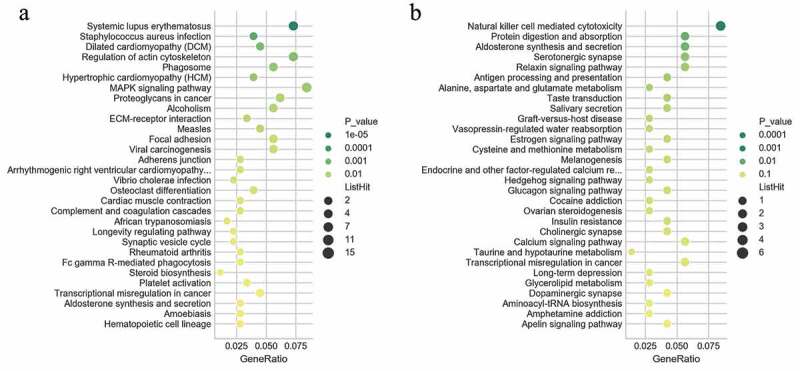
KEGG pathway analysis of differentially expressed genes. (a) KEGG enrichment results of up-regulated and (b) down-regulated differentially expressed genes

The KEGG analysis indicated that the up-regulated mRNAs were mainly enriched in KEGG terms including MAPK signaling pathway, proteoglycans in cancer, ECM-receptor interaction, complement and coagulation cascades, platelet activation, transcription misregulation in cancer, and hematopoietic cell lineage (Figure 4(a,b) and Supplementary Table 3). The downregulated mRNAs were mainly enriched in KEGG terms including natural killer cell mediated cytotoxicity, transcription misregulation in cancer, aminoacyl-Trna biosynthesis, and glycerolipid metabolism (Figure 4(b) and Supplementary Table 3). These items are correlated with pathogenesis and progression of hematological malignancies especially leukemia, which might inform us the direction of following studies in JAK2V617F-positive cMPN. Furthermore, it’s observed that the up-regulated DEGs (Figure 4(a)) and down-regulated DEGs (Figure 4(b)) were enriched in 156 and 229 KEGG terms respectively, of which 132 terms were coincident. Compared with normal controls, JAK2V617F-positive cMPN patients had significantly different gene expression profiles, and the up-regulated and down-regulated DEGs mostly concentrated on the same pathway, suggesting that these differential genes may contribute to the pathogenesis or progression of JAK2V617F-positive cMPNs, which provides a rationale for further in-depth research. It is worth noting that some items, such as systemic lupus erythematosus and rheumatoid arthritis, which are related to autoimmune diseases were also significantly enriched in JAK2V617F-positive cMPN patients’ DEGs and the underlying mechanisms remains to be further studied.

### Functional prediction of the differently expressed lncRNAs through analysis of co-expressed coding genes

**Figure 5.**
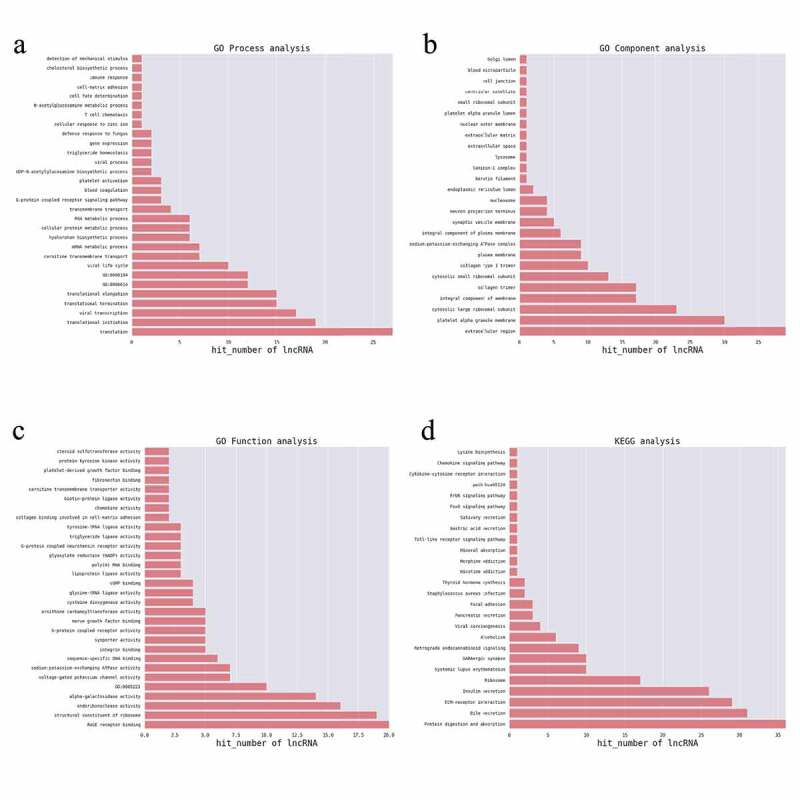
GO and KEGG terms analysis of co-expressed coding genes associated with differentially expressed lncRNAs. The bar demonstrates the degree of enrichment, which was calculated by the following formula: (The number of selected genes in a term/total number of selected genes)/ (the total number of genes in a term of the database/the total number of genes in the database). (a) Biological process; (b) Cellular component; (c) Molecular function; (d) KEGG pathways

To explore potential biological significance of differently expressed lncRNAs, GO and KEGG pathway enrichment were adopted to annotate the co-expressed coding genes of the lncRNAs which were significantly differentially expressed. For each DELs, the co-expressed coding genes were calculated and the hypergeometric distribution test method was used to calculate the significance of the enrichment of coding genes for each GO (or KEGG) entry. A p-value < 0.05 indicated that the differentially expressed co-expressed coding genes was enriched in the GO (or KEGG) entry. GO and KEGG items enriched by genes are shown in Figure 5. The identified genes were enriched mostly in translation, translational initiation, blood coagulation, platelet activation, cell fate determination and RNA metabolic process for BP terms (Figure 5(a)). For CC term analysis (Figure 5(b)), cytosolic large ribosomal subunit, platelet alpha granule membrane, cytosolic small ribosomal subunit, and extracellular region were the significantly enriched components. In terms of the MF analysis (Figure 5(c)), RAGE receptor binding, structural constituent of ribosome and intracellular cGMP activated cation channel activity were important. Furthermore, genes associated with these lncRNAs were mainly enriched in protein digestion and absorption and GABAergic synapses. KEGG pathways (Figure 5(d)) enriched by the DELs-correlated co-expressed coding genes included protein digestion and absorption, bile secretion, extracellular matrix (ECM)-receptor interaction, insulin secretion, ribosome, systemic lupus erythematosus, GABAergic synapse, retrograde endocannabinoid signaling, alcoholism, viral carcinogenesis, pancreatic secretion, and focal adhesion. The blood coagulation [32], platelet activation [33], cell fate determination [34], platelet alpha granule membrane items [35], and protein digestion and absorption pathway [36] were reported to participate in the pathogenesis of MPNs and leukemia, which indicated that the co-expressed coding genes of the lncRNAs which were significantly differentially expressed in JAK2V617F-positive cMPNs are members of the pathogenetic pathways of myeloid neoplasms and the dysregulation of lncRNAs in JAK2V617F-positive cMPNs might play a vital role in pathogenesis of JAK2V617F-positive cMPNs.

### Cis-regulation analysis of DELs in JAK2V617F-positive cMPNs

**Figure 6.**
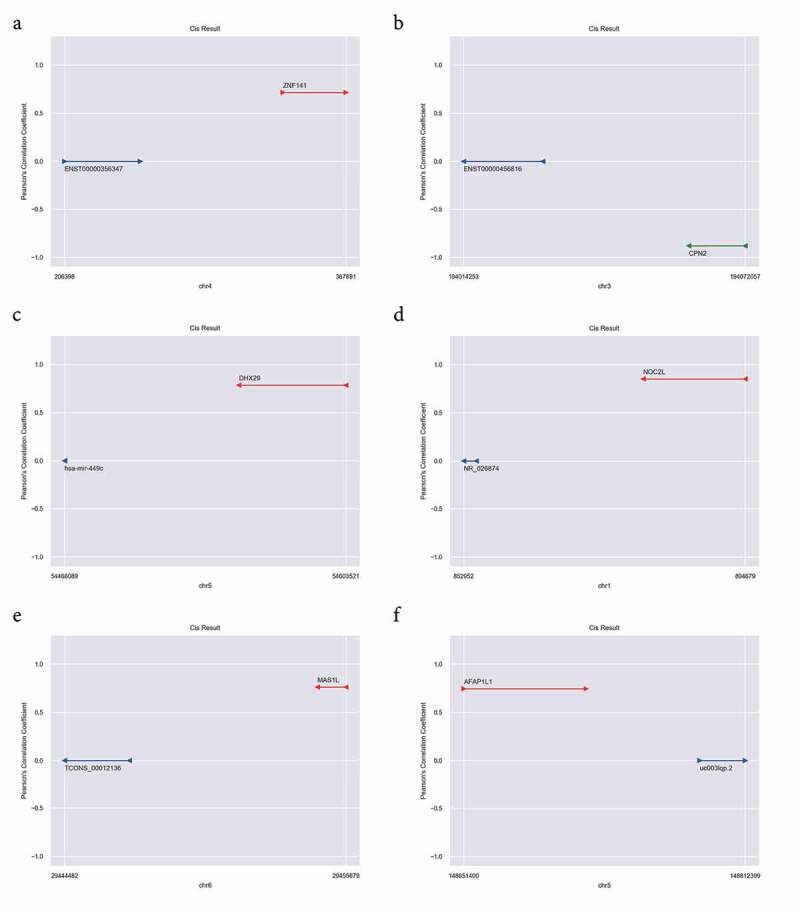
LncRNA Cis-regulation analysis. (a) ENST00000356347 cis-regulates ZNF141; (b) ENST00000456816 cis-results CPN2; (c) hsa-mir-449c cis-results DHX29; (d) NR_026874 cis-results NOC2L; (e) TCONS_00012136 cis-results MAS1L; (f) uc003lqp.2 cis-results AFAP1L1. The red line indicates the genomic position of the mRNA having a positive regulatory relationship with the lncRNA; the green line indicates the genomic position of the mRNA having a negative regulatory relationship with the lncRNA; The blue colored lines mark the genomic position of lncRNAs, and the ordinate is the Pearson’s correlation coefficient between the mRNA and the lncRNA

It has been reported that lncRNAs could be physically associated with the loci surrounding the targeted coding genes, and lncRNAs could act the regulatory function during or immediately following the transcription of these genes via cis-regulation [37]. Cis-regulation analysis (Figure 6) were performed for better understanding the cis-regulation of DELs in JAK2V617F-positive cMPNs. These genes that were adjacent to the genome of identified lncRNAs and co-expressed in their expression patterns were likely to be regulated by the lncRNAs. Through the cis-regulation analysis, we identified six lncRNAs (ENST00000356347, ENST00000456816, hsa-mir-449c, NR_026874, TCONS_00012136, and uc003lqp.2) presenting cis-acting regulation on mRNAs. The results showed that ZNF141, DHX29, NOC2L, MAS1L, and AFAP1L1 were positively cis-regulated by lncRNA ENST00000356347 (Figure 6(a)), hsa-mir-449c (Figure 6(c)), NR_026874 (Figure 6(d)), TCONS_00012136 (Figure 6(e)), and uc003lqp.2 (figure 6(f)), respectively. Furthermore, CPN2 was negatively cis-regulated by ENST00000456816 (Figure 6(b)). These results confirmed that in JAK2V617F-positive cMPN patients, the expression of mRNA was cis-regulated by abnormally-expressed lncRNAs, which further suggested that the dysregulation of lncRNAs might play a role in JAK2V617F-positive cMPN through regulation of the transcription of targeted genes.

### LncRNAs and transcription factor association analysis in JAK2V617F-positive cMPNs

**Figure 7.**
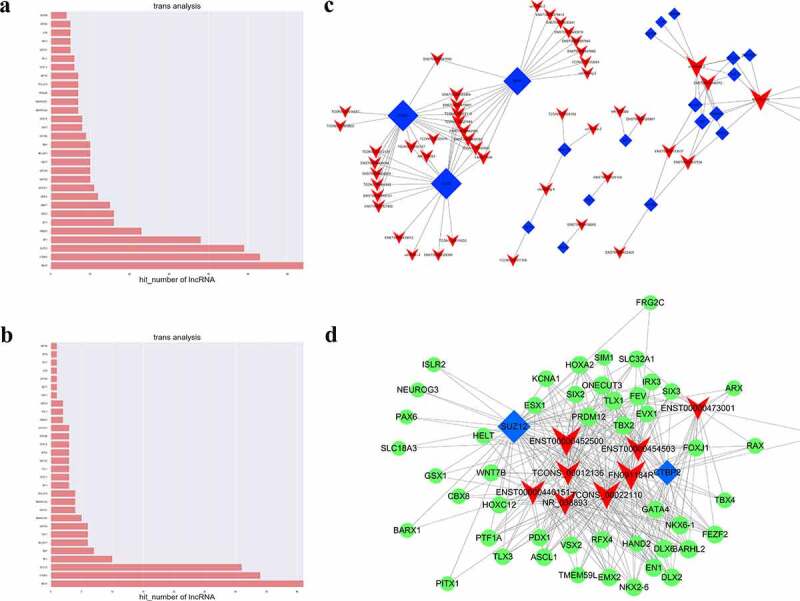
LncRNAs and transcription factor correlation analysis. (a) GO terms enriched in the TFs which significantly associated with DE lncRNAs; (b) KEGG pathways enriched in the TFs which significantly associated with DE lncRNAs; (c) LncRNA-TF pairs enriched in the study; (d) LncRNA-TF relationship pairs and their targeted-differential genes identified the three-element network correlation among lncRNA-TFs-target genes in cMPNs

The intersection of the set of co-expressed mRNAs of lncRNAs with the set of target genes of the transcription factors were calculated in our study to obtain TFs that might be significantly correlated with lncRNAs and to identify TFs that may play a regulatory role together with lncRNAs. The hypergeometric distribution was used to visualize the network mapping. The top 30 differentially expressed mRNAs associated with significantly dysregulated GOs and KEGG pathways were chosen as candidate genes and the enrichment of target genes was calculated (p ≤ 0.05) (Figures 7(a,b) respectively and Supplementary Table 4). We thus determined that TFs might play a role in combination with lncRNAs and might regulate lncRNAs in JAK2V617F-positive cMPNs (Figure 7(c)). Several lncRNA-TF pairs may correlate with one lncRNA, indicating that each lncRNA could function as a regulator with different TFs. These lncRNAs were mostly correlated with CTBP2, SUZ12, REST, STAT2, and GATA4. In the TFs-lncRNA-target genes network, eight DELs with 48 candidate genes and two core TFs (SUZ12 and CTBP2) were identified (Figure 7(d) and Supplementary Table 5). GATA4 was co-expressed with lncRNA ENST00000452500, lncRNA ENST00000454503, NR_038893, lncRNA ENST00000440151, and FN091184R. GATA4 gene was also targeted by CTBP2 and SUZ12. SUZ12 has been speculated to regulate the expression of these eight lncRNAs and its target genes are indicated as the green nodes in Figure 7(d). Finally, based on the lncRNA-mRNA co-expression network, the dysregulated lncRNAs, such as lncRNA ENST00000452500, lncRNA ENST00000473001, lncRNA ENST00000454503, lncRNA TCONS_00012136, and lncRNA NR_038893, as the regulators of PDX1, GATA4, HOXA2, ASCL1, and PAX6, respectively, may play a vital function in JAK2V617F-positive cMPNs. The present networks indicate that there is an inter-regulation relationship between lncRNAs and TFs, and that the regulation between TFs and target genes, as well as the three-element network correlation among lncRNA-TFs-target genes, might participate in the pathogenesis of JAK2V617F-positive cMPNs.

## Discussion

Recently, a growing number of studies have shown that lncRNAs are important components of the human genome that play critical functions. Several studies have reported that dysregulated lncRNAs is associated with cell phenotypes in leukemia. However, what functions lncRNAs play in JAK2V617F-positive cMPNs are still not fully investigated. In this study, we found that JAK2V617F-positive cMPN patients were associated with significant changes of the mRNA and lncRNA expression profiles and the co-expression network of DELs and DEGs. The functional prediction associated with DEGs and DELs in JAK2V617F-positive cMPN patients revealed enrichment in multiple vital pathways related to JAK2V617F-positive cMPN pathogenesis, progression, and prognosis. The cis- and trans-regulation analysis of DELs found that the expression of mRNA were potentially cis-regulated by the abnormally-expressed lncRNAs and the TFs-mRNA were also potentially trans-regulated by DELs in JAK2V617F-positive cMPN, which meant that dysregulation of lncRNAs might play an important role in JAK2V617F-positive cMPN through regulation of the transcription of coding genes and TFs.

A few studies have evaluated the lncRNA profiles of myelodysplastic/juvenile myelomonocytic leukemia patients [38], but few of comprehensive study on the lncRNA profiles of JAK2V617F-positive cMPN patients is available. Our study included samples from patients with JAK2V617F-positive cMPNs and samples from normal controls to perform a genome-wide lncRNA and mRNA expression analysis. According to our microarray analysis, the expression of lncRNAs and mRNAs were significantly dysregulated in JAK2V617F-positive cMPN patients compared with the normal controls (Figure 1). The heatmaps revealed that JAK2V617F-positive cMPN patients have a significant overall difference from normal controls in terms of the expression profiles of coding and long non-coding RNAs (Figures 1(a,b)). We chose DEGs from cMPN-related pathways such as SOCS2, ESAM, FCGR1A, ITGB3, KIR3DL1, and FGF2 and validated their expression in JAK2V617F-positive cMPN patients, and we found that expression of ITGB3 was increased in JAK2V617F-positive cMPN patients compared to their normal controls, while the expression of KIR3DL1 was decreased in JAK2V617F-positive cMPN patients compared to the normal controls (Figure 1(c)). The expression of SOCS2, ESAM, FCGR1A, and FGF2 showed no statistically significant differences between groups, which might be attributed to the small sample size, although the overall trends in expression were consistent with the microarray analysis. Furthermore, the evaluation of ITGB3 transcription and translation levels in a variety of myeloid tumor cell lines revealed that in cell model of JAK2V617F-positive cMPN, the levels of ITGB3 were also far higher than that of negative control cell lines. ITGB3 (β3 integrin), also known as CD61 or GP3A, is a member of the most widely studied integrin family [39]. ITGB3 exerts diverse roles in tumor progression and in the reprogramming of the tumor microenvironment [39]. Moreover, ITGB3 also participates in several blood-related diseases including Glanzmann Thrombasthenia [40] as well as bleeding disorder [41]. GO items related to ITGB3 include identical protein binding and protease binding, cell adhesion molecule binding and platelet-derived growth factor receptor binding, but the role of this gene in cMPNs has not been adequately evaluated to date. Our study initially explored the expression of ITGB3 in JAK2V617F-positive cMPN patients and the results were consistent with the results of GO annotation entries. As ITGB3 gene encodes integrins β3 and form platelet glycoprotein (GP)IIb/IIIa which contributes in fibrinogen, and polycythemia vera characterized with thrombocytosis is one kind of cMPNs, we speculate that ITGB3 may play an important role in JAK2V617F-positive cMPNs and warrants subsequent in-depth study.

Killer Immunoglobulin-like Receptors (KIRs) are receptors expressed on natural killer cells and T-cells [42]. KIR3DL1 is a protein coding gene and is a inhibitory receptor that binds to groups of HLA-A and HLA-B allotypes [43], which plays an important role in viral infections, cancers, autoimmunity, and transplantation [44–46]. KIR3DL1 binding to the BW4 ligand expressed by the HLA-B allotypes transmits inhibitory signals to inhibit the killing effect of NK cells. Previous studies have shown that allelic polymorphisms of KIRs are related to the deep molecular response (DMR) in patients with chronic phase CML (CML-CP) receiving tyrosine kinase inhibitor (TKI) treatment by influencing the NK cell activation status [29,47]. Furthermore, studies have reported that JAK inhibition impairs NK cell function in MPNs [48]. Thus, the functional role of KIRs in JAK2V617F-positive cMPNs still needs further research.

Analysis of the gene co-expression network is of biological significance because the co-expressed genes are controlled by the same transcriptional control program and are functionally related. They are part of the same pathway or protein structure. A set of co-expressed DEGs for every DEL were identified by performing co-expression analysis in our study. We showed heatmaps of the co-expression network of four DELs, the lncRNA_ENST00000414065, lncRNA_ENST00000427852, lncRNA_NR_023926, and lncRNA_ENST00000561476 (Figure 2). The heatmaps showed clearly that four lncRNAs were all correlated with a group of co-expressed DEGs and these co-expressed DEGs were obviously different between JAK2V617F-positive cMPNs patients and normal controls, suggesting that in JAK2V617F-positive cMPNs the DELs had great potential in dysregulating mRNA expression and might take part in the pathogenesis of the disease. The different co-expressed DEGs between JAK2V617F-positive cMPNs patients and normal controls may also suggest potentially targeted biomarkers in JAK2V617F-positive cMPN.

GO and KEGG analyses were performed to predict the function of the DEGs. First, GO analysis annotated the entire set of statistically significant DEGs. It indicated that the DEGs were mostly related to the ‘regulation of cell shape’ in the biological process category, ‘heparin binding’ in the molecular function category, and the ‘integral component of plasma membrane’ in cellular component category (Figures 3(e,f)). The top 10 enriched GO terms shown in Figure 3 implied the main functions the DEGs in the JAK2V617F-positive cMPNs were involved in, which indicated these biological functions might be altered and contribute to the morbidity of JAK2V617F-positive cMPNs. Next, GO analysis was also performed for upregulated and downregulated DEGs subgroups. The results showed that the top 10 enriched GO terms in the three GO categories (biological processes, molecular functions, and cellular components) were almost significantly different between the two subgroups, with the exception of the ‘integral component of plasma membrane’ in cellular component category (Figures 3(a,c)). This latter category was the most enriched term in the downregulated DEGs subgroup (Figure 3(d)) and ranked third in the upregulated DEGs subgroup (Figure 3(b)). These results indicated that the DEGs involved in the ‘integral component of plasma membrane’ biological function might play an important role in the pathogenesis of JAK2V617F-positive cMPNs. Comparing the images in Figure 3, we observed that the top 10 enriched GO terms in the three GO categories were the same as those of the entire dataset and for the upregulated subgroup. This might because most DEGs in the dataset were upregulated; thus, the upregulated subgroup exhibited more weight and was responsible for a larger proportion of the effect, although the upregulated DEGs may also have had a greater influence on the pathogenesis of JAK2V617F-positive cMPNs than the downregulated DEGs. The KEGG analysis was performed and revealed the entire dataset of DEGs was mainly enriched in KEGG terms including ‘natural killer cell mediated cytotoxicity’, ‘protein digestion and absorption’, ‘aldosterone synthesis and secretion’, ‘relaxin signaling pathway’, ‘*Staphylococcus aureus* infection’, ‘MAPK signaling pathway’, ‘complement and coagulation cascades’, and ‘systemic lupus erythematosus’. The downregulated DEGs subgroup was mainly enriched in KEGG terms including ‘NK cell mediated cytotoxicity’, ‘protein digestion and absorption’, ‘aldosterone synthesis and secretion’, and ‘relaxin signaling pathway’. The aberrantly upregulated mRNAs subset was mainly enriched in KEGG terms including *Staphylococcus aureus* infection and the MAPK signaling pathway (Figure 4). Some of these pathways were reported to regulate the pathogenesis of leukemia, but their roles in JAK2V617F-positive cMPNs still require further research. The DEGs largely concentrated on the same pathway, suggesting that these differential genes may be related to the onset or progression of JAK2V617F-positive cMPNs, which warrants more in-depth research. The reason why certain pathways not related to hematological tumors, such as ‘Staphylococcus aureus infection’ and ‘systemic lupus erythematosus’, are enriched in JAK2V617F-positive cMPN patients remains to be further studied, which might be an instructive direction.

We also performed the GO and KEGG analyses to predict the functional role of the DELs. Many genes were screened that were co-expressed with DELs and these were mainly involved in translation, translational initiation, blood coagulation, platelet activation, cell fate determination, RNA metabolic processes, the platelet alpha granule membrane, cytosolic large ribosomal subunit, RAGE receptor binding, structural constituent of ribosome and intracellular cGMP activated cation channel activity, protein digestion and absorption, and the GABAergic synapse (Figure 5). Thrombosis is a vital cause of mortality in cMPN patients, which involves multiple cellular mechanisms including platelet activation [49]. Our study found that JAK2V617F-positive cMPN patients-related DELs were involved in blood coagulation, platelet activation and platelet alpha granule membrane, indicating that platelet-related pathways play a vital role in the pathogenesis and progression of JAK2V617F-positive cMPNs, which need to be further explored. It’s reported that blockade of the RAGE increases S100A8/9-mediated inhibition of AKT signaling regulated by JAK2V617F mutant allele burden [50]. Our study indicated that JAK2V617F-positive cMPN patients-related DELs were involved in the RAGE receptor binding pathway. Further research might shed light on the diagnosis and treatment of JAK2V617F-positive cMPNs. These studies showed that the dysregulated lncRNAs may play a role in JAK2V617F-positive cMPNs through multiple pathways.

LncRNA expression is associated with adjacent (cis-regulation) or distal (trans-regulation) protein-coding genes, which contributes to gene expression regulation and genome complexity in human cancer [51]. Thus, we performed the analysis of cis-regulated mRNAs and trans-regulated mRNAs to further annotate the regulation function of lncRNAs in JAK2V617F-positive cMPN. The cis-regulation analysis of the DELs revealed that ZNF141, DHX29, NOC2L, MAS1L, and AFAP1L1 were positively cis-regulated by lncRNA ENST00000356347, hsa-mir-449c, NR_026874, TCONS_00012136, and uc003lqp.2, respectively. Instead, CPN2 was negatively cis-regulated by ENST00000456816 (Figure 6). ZNF141 is a protein-coding gene that encodes a zinc finger protein and exerts oncogenic effects, and defects in this gene contributes to the pathogenesis of autosomal recessive postaxial polydactyly type A [52]. DHX29 encodes a protein functions in translation initiation, and knockdown of DHX29 reduces protein translation and impaired cancer cells proliferation. Histone acetyltransferases (HAT) and histone deacetylases (HDAC) act as a role of transcriptional regulation by histone modification [53], and NOC2L is a novel HDAC-independent inhibitor of HAT. Overall, our analysis showed that lncRNA exhibits transcriptional activation and expression regulation on adjacent mRNA, and this regulation of gene expression may be associated with the pathogenesis and development of JAK2V617F-positive cMPNs [54]. These results might provide a rationale for further in-depth research.

LncRNA trans-regulation involves the downstream regulation of mRNA transcription including distant genes such as binding enhancers and promoters [55] and lncRNAs regulate the activity of the bound protein or mRNA in a dose-dependent manner in the cytoplasm or nucleus [56]. Through the trans-regulation analysis of the DELs, we found that 23 TFs had been predicted to regulate the DELs. The TFs-DELs-target genes network identified by our data analysis included eight DELs, 48 co-expressed DEGs, and two core TFs (SUZ12 and CtBP2). It has been reported that SUZ12 inhibition and mutant JAK3 plays a synergistic effect to promote T-cell transformation and T-cell ALL [57]. Aberrant expression of CtBP2 has been observed in ovarian cancer, melanoma, breast cancer, and esophageal squamous cell carcinoma [58]. CtBP2 also promotes the de novo methylation in pediatric B-cell-ALLs [59]. The DEGs in the TFs-DELs-target genes network included genes such as HOXA2, GATA4, PRDM12, SLCs, TBX2, and PAX6. The Hox genes encode TFs that control cellular differentiation and development [60], and the dysregulation of Hox genes plays a key role in acute myeloid leukemia (AML) [61]. It’s reported that in pediatric AML patients, GATA4 was epigenetically inactivated by promoter hypermethylation, and acts as a tumor suppressor [62]. PRDM12 gene is a member of the PR-domain-containing zinc-finger family. It functions as a negative regulator of oncogenesis and has been reported as a third partner gene in CML [63]. The deregulation of this gene is associated with solid cancers and hematological malignancies including CML [64]. The solute-carrier gene (SLC) superfamily encodes membrane-bound transporters [65], and there are significant differences in the expression of SLC genes between tumor and healthy tissue and the dysfunction of SLCs will disrupt homeostasis [66]. TBX2, a member of the T-box transcription factor family, is overexpressed in several cancers and may have a potential role in tumorigenesis as an immortalizing agent [67,68]. PAX6 belongs to a family of genes that play a critical role in the tissues and organs formation during embryonic development [69] and are also important for maintaining the normal function of certain cells [70]. It has been reported that PAX6 networked with the LncRNA DANCR and played a key regulatory role in apoptosis and autophagy of breast cancer cells [71]. In summary, the lncRNA trans-regulation analysis of our study identified the TFs-DELs-target genes network showed that lncRNAs play a regulatory role in combination with transcription factors or chromatin regulators, and the lncRNA-TFs relationship pairs regulated multiple target genes to contribute to the etiology and progression of JAK2V617F-positive cMPNs. Furthermore, these studies indicated that these lncRNAs and mRNAs differentially expressed in JAK2V617F-positive patients are closely related to hematological tumors, and suggests they may also be associated with the pathogenesis of JAK2V617F-positive cMPNs, features which warrant further in-depth research.

This study has several limitations. Firstly, clinical significance including prognosis and clinical correlation of the DEGs and DELs were not explored in our study because of the lack of enough public databases on JAK2V617F-positive cMPNs. Secondly, the functions of some important DEGs or DELs were not investigated to elucidate the role and mechanism of these genes in JAK2V617F-positive cMPNs. Thirdly, all the cMPN patients included in our study were JAK2V617F-positive, and differences in expression profiles between JAK2V617F-negative patients and normal controls, as well as differences in expression profiles between JAK2V617F-positive and JAK2V617F-negative patients still need to be explored by collecting more patient specimens. Finally, as an important element of non-coding RNA, miRNA also plays an important role. The networks established in our study didn’t cover the potential role of miRNA in JAK2V617F-positive cMPNs. Further research focusing on these aspects will carry out and our subsequent in-depth study will follow up persistently.

### Conclusions

In summary, our analysis provides comprehensive novel knowledge on the role of mRNAs and lncRNAs in JAK2V617F-positive cMPNs. The expression profiles of lncRNAs and mRNAs were significantly dysregulated in JAK2V617F-positive cMPNs patients compared with the normal controls, and that ITGB3 might be an important carcinogenic factor. A set of co-expressed DEGs were identified for every DEL, and determined co-expressed DEGs were cis-regulated by several lncRNAs. Finally, a TFs-DELs-target genes network including two core TFs, eight DELs and forty-eight co-expressed target genes was constructed. Our findings illustrated the molecular changes in JAK2V617F-positive cMPNs patients and the potential TFs driving this alteration.

## Supplementary Material

Supplemental Material

## Data Availability

The datasets used and/or analyzed during the current study are available from the corresponding author on reasonable request.

## Research highlights

- Expression profiles of lncRNAs and mRNAs are dysregulated in JAK2V617F-positive cMPN.
- ITGB3 expression is elevated in JAK2V617F-positive cMPN.
- Co-expressed mRNAs are cis-regulated by lncRNAs in JAK2V617F-positive cMPN.
- The lncRNAs-TFs-mRNAs networks were identified in JAK2V617F-positive cMPN.
